# *Aspergillus fumigatus* biofilm formation on different bone substitutes used in maxillary sinus augmentation: an in vitro analysis

**DOI:** 10.1186/s40729-019-0175-5

**Published:** 2019-06-20

**Authors:** Claudio Stacchi, Veronica Del Lupo, Federico Berton, Teresa Lombardi, Raffaela Bressan, Roberto Di Lenarda, Cristina Lagatolla

**Affiliations:** 1 Department of Medical, Surgical and Health Sciences, University of Trieste, Trieste, Italy; 2 Cassano allo Ionio, Italy; 3 Department of Life Sciences, University of Trieste, Trieste, Italy

## Abstract

**Background:**

Fungus ball (FB) typically affects healthy adults, and *Aspergillus fumigatus* is the most frequent etiologic agent: iatrogenic factors represent an important issue in FB pathogenesis. Moreover, a recent study suggested a significant association between the use of anorganic bovine bone as sinus grafting material and subsequent development of FB. The aim of the present investigation is to evaluate in vitro eventual differences in the ability of *Aspergillus fumigatus* to colonize different bone grafting materials and grow on them as biofilm.

**Findings:**

Five different bone substitutes (demineralized bone matrix, anorganic bovine bone, ß-tricalcium phosphate, synthetic nano-hydroxyapatite, and synthetic hydroxyapatite), commonly used in sinus floor augmentation procedures, were inoculated with conidia suspensions of *A. fumigatus* and incubated at 37 °C for 4 and 8 h, in standardized conditions. Biofilm bound to the different materials underwent quantitative and qualitative analysis by confocal and scanning electron microscopy. *A. fumigatus* proved to be able to adhere and form biofilm on all the tested bone substitutes. The surface plot representation of the samples displayed some differences in the density of the superficial layer, due to the physical characteristics of the biomaterials. Nevertheless, Kruskal–Wallis test showed no significant differences in biomass amount among the five bone substitutes (*p* = 0.236 and *p* = 0.55 after 4 and 8 h adhesion, respectively).

**Conclusions:**

All the bone substitutes normally used in sinus floor augmentation represent a favorable substrate for fungal growth, due to their physical and chemical characteristics. During sinus floor elevation procedures, Schneiderian membrane integrity should be maintained in order to avoid the exposure of the grafting material at the respiratory environment, with potential risks of fungal colonization.

## Introduction

*Aspergillus fumigatus* is a filamentous fungus of the *Ascomycetes* class, which represents the most common *Aspergillus* species causing disease in immunocompromised hosts. It is widespread in nature and very common in the environment, in soil, and in decaying organic matter. Colonies of the fungus produce thousands of minute spores called conidia with a diameter of 2–3 μm, presenting a highly hydrophobic surface that allows them to remain in air for prolonged periods [1].

Spores are normally inhaled, but in healthy individuals, they are quickly eliminated by the alveolar macrophages, the major resident phagocytic cells in the respiratory tract. If spores are not eliminated (typically in immunocompromised patients), a process of isotropic growth may be activated, with a rapid proliferation in hyphae spreading in the body and possibly leading to hematogenous dissemination [2].

Plaignaud reported in 1791 the first case of fungal sinusitis [3]: since then, cases of non-invasive extramucosal fungal infection involving the paranasal sinuses (fungus ball) have been reported with increasing frequency; data show that 10 to 30% of all patients with chronic sinusitis present an *Aspergillus* infection [4–6].

Fungus ball (FB) typically affects healthy adults, and *Aspergillus fumigatus* is the most frequent etiologic agent in European and North American patients. Iatrogenic factors represent an important issue in FB pathogenesis: it was demonstrated that the presence of zinc oxide endodontic sealers into the maxillary sinus is the most commonly recognized local risk factor for the disease development [4–7]. Nowadays, it is well known that zinc promotes fungal growth as it represents an essential microelement for helping fungi to survive and proliferate [8, 9].

Furthermore, a recent case-control study suggested a significant association between the use of a specific bone substitute (anorganic bovine bone) as sinus grafting material and subsequent development of FB [10]. Although both lateral and transcrestal sinus floor elevation have been reported as predictable and reliable techniques [11, 12], they have also been associated with several postoperative complications [13, 14], such as graft infection both from fungal and bacterial pathogens [10, 15]. When a biomaterial is grafted in the maxillary sinus, especially in case of Schneiderian membrane tearing or perforation, it may get in contact with the respiratory environment. Moreover, bone substitutes could provide an ideal substrate for fungal adhesion and proliferation: the porous nature of these biomaterials could favor fungus colonization by making it inaccessible to immune surveillance and defensive mechanisms [16].

As the pathogenetic pathways of the fungal infection in the maxillary sinus are still partially unknown, and it was hypothesized a pathophysiological role for a specific bone substitute, the aim of the present in vitro study was to evaluate eventual differences in the ability of *Aspergillus fumigatus* to colonize different bone grafting materials and grow on them as biofilm, a structured community of microorganisms enclosed within a protective extracellular matrix.

## Materials and methods

### Strain and growth conditions

Conidia suspensions of *A. fumigatus* NCPF 7367, obtained from Public Health England Culture Collections, were prepared as described by Mowat et al. [17] Briefly, the strain was streaked out from glycerol stocks stored at − 80 °C and incubated at 37 °C for 3 days. Conidia were harvested by flooding the surface of the plates with 5 ml of phosphate-buffered saline (PBS) containing 0.025% (*v*/*v*) Tween-20 (Sigma Aldrich, St. Louis, MO, USA) and rocking gently, washed in the same buffer, counted using a Burker hemocytometer, and diluted to a density of 10^5^ spores/ml in 3-(N-morpholino) propanesulfonic acid (MOPS)-buffered Roswell Park Memorial Institute medium (RPMI) 1640 (Sigma Aldrich, St. Louis, MO, USA), supplemented with 10% fetal bovine serum (FCS). This suspension was used as inoculum.

### Biofilm formation

Five different bone substitutes, commonly used in sinus floor augmentation procedures, were selected for the experiment. The biomaterials included one allograft (demineralized bone matrix, Puros, Zimmer Biomet, Palm Beach Gardens, FL, US), one xenograft (anorganic bovine bone, BioOss, Geistlich, Wolhusen, Switzerland), and three alloplastic materials (ß-tricalcium phosphate, ßTCP, RTR, Septodont, Saint Maur des Fossés, France; synthetic nano-hydroxyapatite, NHA, Fisiograft Bone, Ghimas, Casalecchio di Reno, Italy; synthetic hydroxyapatite, HA Idrossilapatite, Centro di Odontoiatria Operativa, Padova, Italy). About 50 mg of each bone substitute was distributed in a 24-well microtiter plate, inoculated with 0.5 ml of the conidia suspension, and incubated at 37 °C with orbital shaking (130 rpm) for 4 and 8 h, to allow the attachment of the fungal cells. After these contact periods, the materials were washed to remove the unbound cells, and 1 ml of fresh RPMI medium was added to allow the growth of the biofilm for further 20 h. Finally, samples were thoroughly washed three times with PBS.

### Confocal microscopy analysis

Biofilm bound to the different materials was stained for polysaccharides with 1 ml of 25 μg/ml concanavalin A-Texas Red conjugated (CATR) (Molecular Probes, Eugene, OR, US) for 30 min in static conditions, gently washed once with water and examined by confocal microscopy (CLSM) using a Nikon C1-SI confocal microscope (Nikon Instruments Europe BV, Amsterdam, Netherlands). Excitation and emission wavelength were set to 561 and 600 nm, respectively. Z-stacks were acquired with a 20x/0.5 air objective at 2 μm intervals. The image stacks collected by CSLM were analyzed with the EZ-C1 Free Viewer (Nikon Corporation, Tokyo, Japan) and the Image J 1.47 (Wayne Resband, National Institutes of Health, Bethesda, MD, USA) softwares.

Twelve images, randomly acquired from three independent experiments for each time of adhesion, were analyzed by the COMSTAT software package (www.comstat.dk) to evaluate the amount of the biomass [18].

### Scanning electron microscope analysis

Specimens were fixed at 60 °C for 2 h, then mounted on aluminum stubs covered with two-sided conductive carbon adhesive tape. Subsequently, the samples were sputtered with gold (Sputter Coater K550X, Emitech, Quorum Technologies, Lewes, UK) and immediately analyzed by means of a scanning electron microscope (SEM) (Quanta250 SEM, FEI, Hillsboro, OR, US) operated in secondary electron detection mode. The working distance was adjusted in order to obtain the suitable magnification; the accelerating voltage was set to 30 kV.

### Statistical analysis

Statistical analysis was performed by means of SPSS 15.0 software (SPSS, Chicago, IL, US). Data for descriptive statistics were expressed as mean ± SD. The normality of the distribution and the equality of variance were assessed with Kolmogorov–Smirnov and Levene test, respectively. Data were then analyzed with Kruskal–Wallis test, and statistical significance was pre-set at *p* < 0.05.

## Results

### Qualitative analysis

**Fig. 1:**
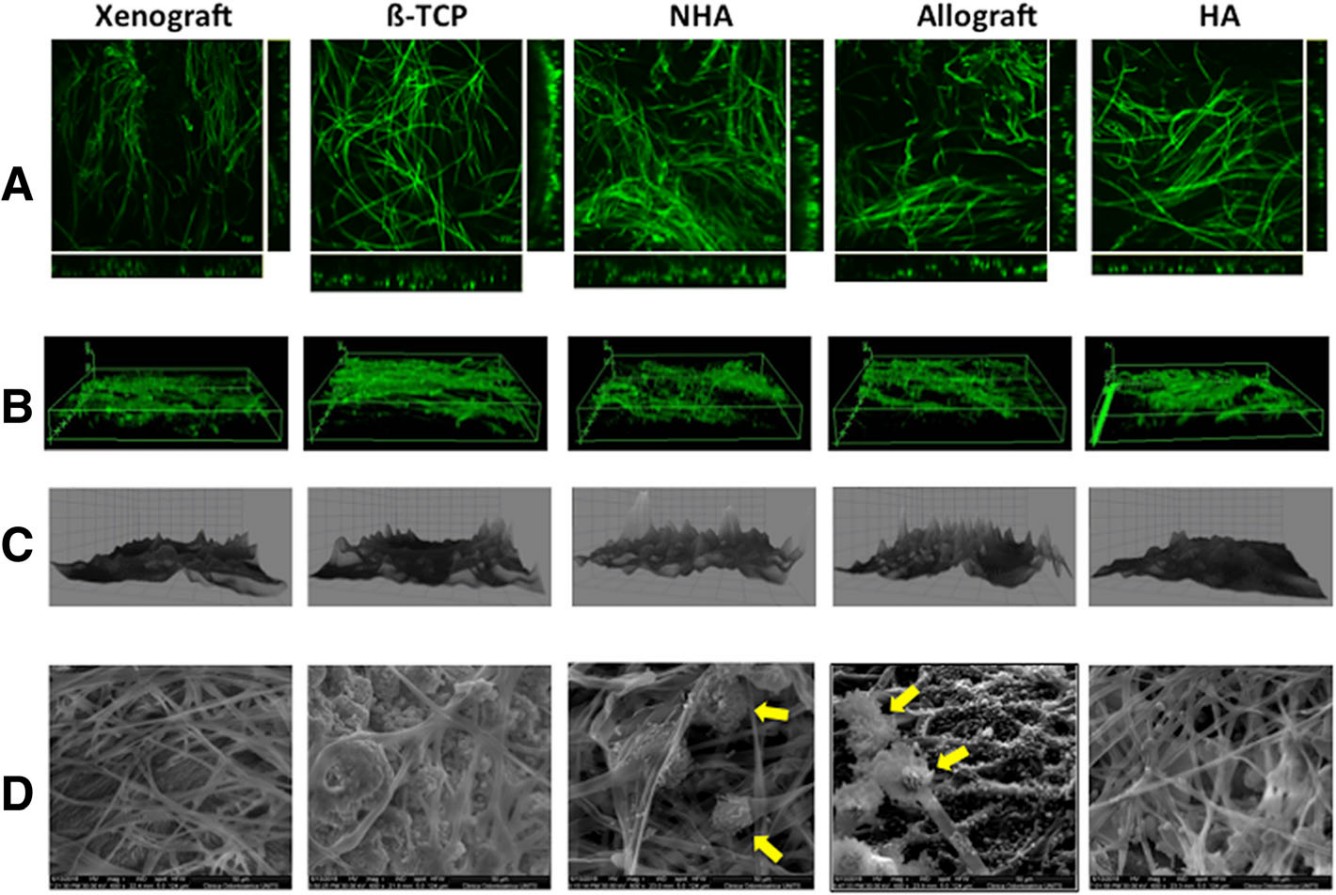
CLSM and SEM micrographs of the *A. fumigatus* biofilm on five different bone substitutes. Xenograft: anorganic bovine bone; ß-TCP: ß-tricalcium phosphate; NHA: synthetic nano-hydroxyapatite; Allograft: demineralized bone matrix; HA: synthetic hydroxyapatite. 10^5^ conidia/ml inoculated onto the different materials were incubated for 8 h to allow adhesion. After washing the unbound cells, the biofilm was grown in fresh medium for 20 h, washed gently with PBS and analyzed with CLSM (**a**, **b**, **c**) and SEM (**d**). **a** Orthogonal view, showing the mycelium morphology, characterized by highly intertwined hyphae. **b** 3D volume image, displaying the biofilm pellicle distributed over the surface of the materials. **c** 3D surface plot, revealing the lower density of the superficial layer of the biofilm formed on NHA and allograft. **d** × 600 magnification SEM micrographs: conidiophore detected in the biofilm formed on NHA and allograft are indicated by yellow arrows

*A. fumigatus* NCPF 7367 proved to be able to adhere and form biofilm on all the tested bone substitutes. Concanavalin staining revealed the presence of highly intertwined superficial hyphae (Fig. 1a) distributed over the surface of the different materials (Fig. 1b). The surface plot representation (Fig. 1c) displayed some differences in the density of the superficial layer, which looked rather wispy in the biofilm formed on NHA and allograft compared to that detected on the other samples. This property was confirmed by SEM analysis that, besides the filamentous hyphae detected in all samples, revealed the presence of conidiophora only on the surface of NHA and allograft (Fig. 1d).

### Quantitative analysis

**Fig. 2:**
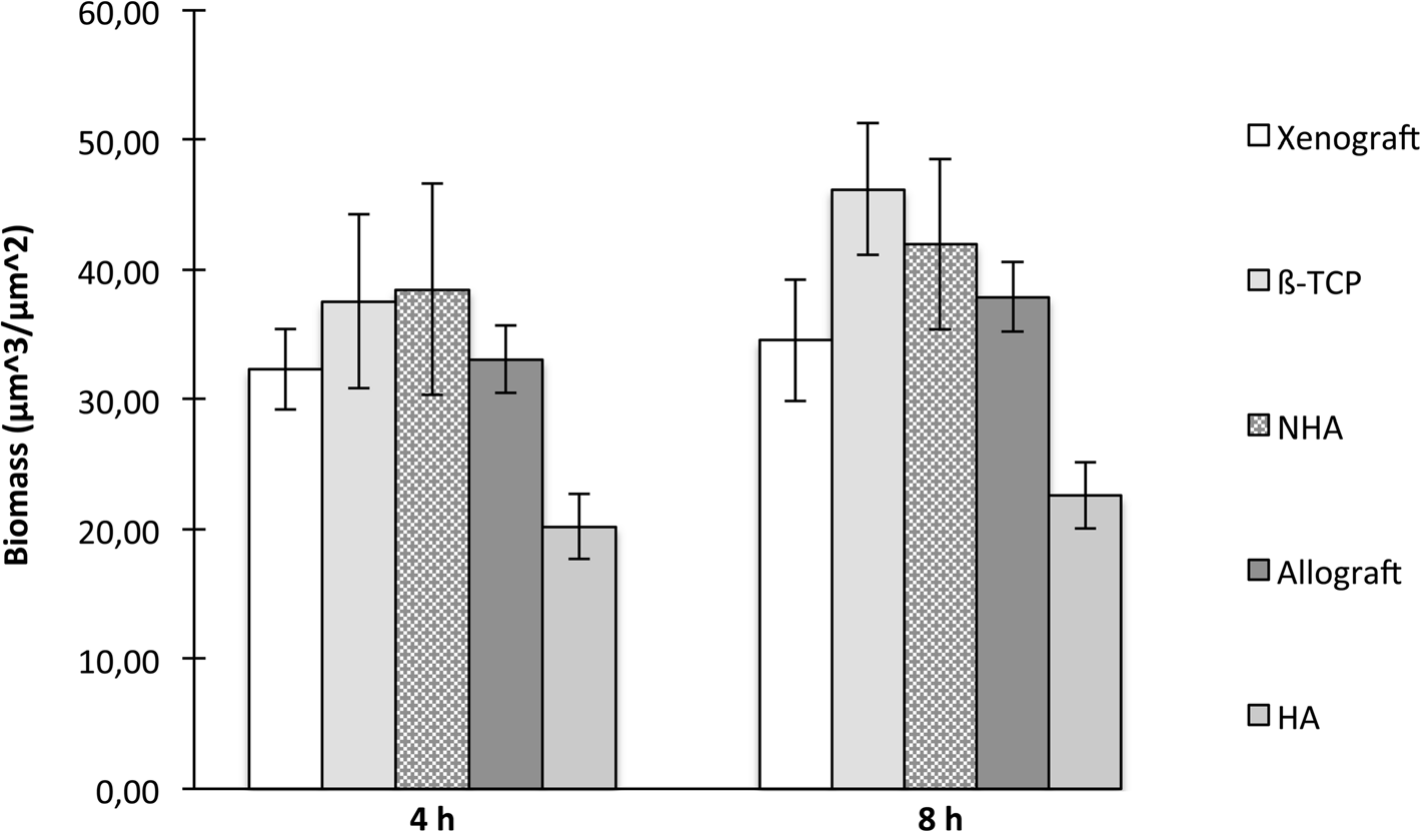
Quantitative analysis of the biofilm formed by *A. fumigatus* on five different bone substitutes. Xenograft: anorganic bovine bone; ß-TCP: ß-tricalcium phosphate; NHA: synthetic nano-hydroxyapatite; Allograft: demineralized bone matrix; HA: synthetic hydroxyapatite. Biomass evaluation was assessed in a 20-h-old biofilm produced by conidia tightly bound to the bone substitutes after 4 and 8 h adhesion times. For each sample, twelve confocal laser scanning microscope (CLSM) image stacks were acquired at random positions and the biomass was quantified by COMSTAT analysis. Results are expressed as the mean values among 12 CLSM image stacks ± SD, represented by the error bars. For each adhesion time, comparison between materials was made with the Kruskal–Wallis test for non-normally distributed data: *p* values are reported above

The evaluation of the biomass formed on each bone substitute, obtained by COMSTAT analysis and expressed as the mean values ± SD from twelve images randomly acquired, showed small differences among the five materials (Fig. 2). However, these differences resulted not significant after statistical analysis by the Kruskal–Wallis test, as *p* values obtained for both experimental conditions (4 and 8 h adhesion before washing unbound cells) were 0.236 and 0.55, respectively.

## Discussion

The pathophysiology of fungus ball (FB) remains not completely understood, even if two theories have been formulated to explain it [5]. The aerogenic theory postulates that inhaled fungal spores which were not cleared by mucociliary system in a maxillary sinus with impaired homeostasis could develop into a FB. The iatrogenic theory hypothesizes that fungal colonization of the maxillary sinus occurs via oroantral communications after dental treatment. The combination of dentogenic factors, such as endodontic treatments, extractions, or apical periodontitis in teeth with the apex in strict contiguity with the sinus floor, were shown to correlate significantly with FB development [19]. In particular, zinc oxide-based endodontic sealers have been demonstrated to promote fungal growth [8, 9, 20], and their protrusion into the sinusal cavity, due to root canal overfilling, may significantly contribute to the onset of mycotic infections [21].

During sinus augmentation procedures, bone substitutes are normally grafted in the space created between the sinus floor and Schneiderian membrane, after its careful detachment and elevation from the bony walls. In case of membrane perforation or tearing, an intra-operative complication occurring with an incidence varying from 5 to 20% depending on the surgical technique [22], a dissemination of the biomaterial in the maxillary sinus cavity is a possible event. Bone substitute granules in contact with the respiratory environment could be easily colonized by resident fungi, also considering that a temporary impairment of the mucociliary clearance always occurs after sinus surgery [23]: nevertheless, very few studies were published on this topic [10, 16].

In a recent retrospective study, Scolozzi et al. hypothesized the physiopathological role of a bone substitute, by demonstrating a significant association between a specific anorganic bovine bone (BioOss, Geistlich, Wolhusen, Switzerland) and subsequent development of FB [10]. The results of the present study, testing five different biomaterials of three different classes (allografts, xenografts, and alloplasts), do not support this finding by showing no significant differences among the various grafts in promoting adhesion and biofilm formation of *A. fumigatus*. All the bone substitutes here tested represented an ideal substrate for fungal growth: their interconnected porosities greatly favored *A. fumigatus* adhesion and proliferation [24]. In fact, the colonization of the substrate was very rapid with all the investigated biomaterials: 8 h adhesion resulted only in a slight increase in the amount of the biomass compared to the samples incubated for 4 h before washing unbound cells, indicating that 4 h is a sufficient time for *A. fumigatus* to effectively adhere to all the materials of the test, as previously suggested [25].

The qualitative analysis revealed the presence of highly intertwined superficial hyphae on the surface of all the different biomaterials, but the superficial layer of NHA and allograft exhibited some differences from the other bone substitutes. Besides the filamentous mycelium detected in all samples, SEM analysis showed the presence of conidiophora only on the surface of NHA and allograft. A possible explanation for the formation of an aerial mycelium only on these two biomaterials can be related to their lower density compared to the other bone substitutes here tested. In fact, it has been shown that filamentous fungi growing at air-water interfaces produce some small hydrophobic proteins, named hydrophobins, which play important roles in the switching towards aerial hyphae formation and spore production [26]. Taking into account that both allograft and NHA floated on the surface of the liquid medium during incubation, while xenograft, ß-TCP, and HA, being heavier, remained completely submerged, the different growth pattern of *A. fumigatus* on the various biomaterials could be likely ascribed to more or less aerobic growth conditions.

In conclusion, the results of the present study showed that all the bone substitutes normally used in sinus floor augmentation represent a favorable substrate for fungal growth, due to their physical and chemical characteristics. During sinus floor elevation procedures, Schneiderian membrane integrity should be maintained by using proper surgical approaches [27, 28], in order to avoid the exposure of the grafting material at the respiratory environment, with potential risks of fungal colonization.

## Abbreviations

CATR: Concanavalin A-Texas Red
CLSM: Confocal laser scanning microscopy
FB: Fungus ball
FCS: Fetal bovine serum
HA: Synthetic hydroxyapatite
MOPS: 3-(N-morpholino) propanesulfonic acid
NHA: Synthetic nano-hydroxyapatite
PBS: Phosphate-buffered saline
RPMI: Roswell Park Memorial Institute medium
SEM: Scanning electron microscopy
ß-TCP: ß-Tricalcium phosphate
